# Temperature‐dependent dynamic control of the TCA cycle increases volumetric productivity of itaconic acid production by *Escherichia coli*

**DOI:** 10.1002/bit.26446

**Published:** 2017-10-06

**Authors:** Björn‐Johannes Harder, Katja Bettenbrock, Steffen Klamt

**Affiliations:** ^1^ Max Planck Institute for Dynamics of Complex Technical Systems Magdeburg Germany

**Keywords:** dynamic metabolic control, *Escherichia coli*, genetic switch, itaconic acid production, two‐stage process, volumetric productivity

## Abstract

Based on the recently constructed *Escherichia coli* itaconic acid production strain ita23, we aimed to improve the productivity by applying a two‐stage process strategy with decoupled production of biomass and itaconic acid. We constructed a strain ita32 (MG1655 Δ*aceA* Δ*pta* Δ*pykF* Δ*pykA* pCadCs), which, in contrast to ita23, has an active tricarboxylic acid (TCA) cycle and a fast growth rate of 0.52 hr^−1^ at 37°C, thus representing an ideal phenotype for the first stage, the growth phase. Subsequently we implemented a synthetic genetic control allowing the downregulation of the TCA cycle and thus the switch from growth to itaconic acid production in the second stage. The promoter of the isocitrate dehydrogenase was replaced by the Lambda promoter (*p*_R_) and its expression was controlled by the temperature‐sensitive repressor *CI857* which is active at lower temperatures (30°C). With glucose as substrate, the respective strain ita36A grew with a fast growth rate at 37°C and switched to production of itaconic acid at 28°C. To study the impact of the process strategy on productivity, we performed one‐stage and two‐stage bioreactor cultivations. The two‐stage process enabled fast formation of biomass resulting in improved peak productivity of 0.86 g/L/hr (+48%) and volumetric productivity of 0.39 g/L/hr (+22%) in comparison to the one‐stage process. With our dynamic production strain, we also resolved the glutamate auxotrophy of ita23 and increased the itaconic acid titer to 47 g/L. The temperature‐dependent activation of gene expression by the Lambda promoters (*p*_R_/*p*_L_) has been frequently used to improve protein or, in a few cases, metabolite production in two‐stage processes. Here we demonstrate that the system can be as well used in the opposite direction to selectively knock‐down an essential gene (*icd*) in *E. coli* to design a two‐stage process for improved volumetric productivity. The control by temperature avoids expensive inducers and has the potential to be generally used to improve cell factory performance.

## 1 INTRODUCTION

Due to environmental concerns, the sustainable production of chemicals by microbial fermentation has attracted an increasing interest in recent years. To become competitive with traditional chemical processes, bio‐based processes for bulk chemicals should have a minimum yield of 80% of the theoretical yield, a titer of 50 g/L and a volumetric productivity of around 3 g/L/hr (Van Dien, 2013; Werpy & Petersen, 2004). Improvements of yield and titer by genetic engineering or process optimization have been realized for a wide range of products including amino acids, organic acids, or biofuels, however, the volumetric productivity remained low in most cases (Becker & Wittmann, 2015). Reasons for low volumetric productivities are the competition between biomass and product synthesis for central metabolic precursors and, depending on the product, also growth inhibition by toxic products or intermediates. Beside the optimization of flux distributions for increased biomass formation by adaptive evolution or targeted over‐expression, the decoupling of biomass production and product formation in two‐stage processes has the potential to increase volumetric productivities.

The separation of biomass and product formation is commonly used for protein production (induced in second stage), production of secondary metabolites (naturally induced in stationary phase), or product processes induced by nutrient limitations. Also the production of biomass in an aerobic phase followed by a micro‐aerobic or anaerobic production phase is suitable for some reduced products including succinate (Beauprez, De Mey, & Soetaert, 2010) or lactate (Zhou et al., 2010). With the arising progress in genetic engineering, the development of molecular devices that dynamically switch genes on or off has become a new and attractive approach for establishing synthetic two‐stage production processes for a wide range of products. Different strategies have been considered and are reviewed in (Burg et al., 2016; Venayak, Anesiadis, Cluett, & Mahadevan, 2015). For industrial processes, it is important to choose a switching mechanism that is triggered, at the right time point, by intracellular or external (process) signals avoiding expensive inducers. Whereas several dynamic genetic switches have been reported that activate the expression of certain genes, only a few approaches have been realized to switch off the expression of certain genes, for example, via environmental signals like quorum sensing (Gupta, Reizman, Reisch, & Prather, 2017) or temperature (Cho, Seo, Kim, Jung, & Park, 2012).

**Figure 1:**
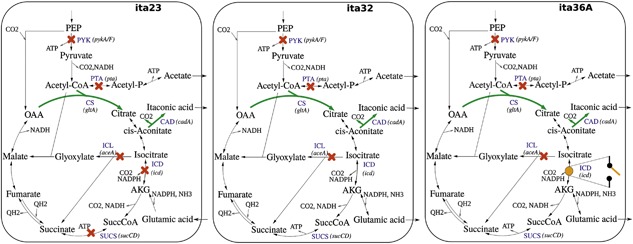
Schematic representation of the metabolic networks of strains ita23, ita32, and ita36A. Green bold line: upregulation of gene; red crosses: gene deletions, or (in case of *icd* in ita23) strong downregulations. Orange point (ita36A): temperature‐dependent regulation

Recently, we reported the model‐based construction of a high‐yield itaconic acid production strain of *Escherichia coli* (ita23) based on static interventions enforcing a coupling of growth and product synthesis (Harder, Bettenbrock, & Klamt, 2016; see Figure 1 left). The constructed strain ita23 produced itaconic acid in a fed‐batch process with an exceptionally high yield (0.68 mol/mol) and titer (32 g/L) and with a maximum volumetric productivity of 0.45 g/L/hr. To further increase the productivity, and to avoid glutamate auxotrophy of this strain during growth, in this work we aimed to dynamically control the flux through the tricarboxylic acid (TCA) cycle to allow for a high TCA cycle flux in the growth phase and a low TCA cycle flux for maximal itaconic acid synthesis in the production phase. We decided to control the TCA cycle by temperature‐dependent expression of the isocitrate dehydrogenase gene (*icd*). We first implemented the dynamic control of *icd* into the wild type and proved the feasibility of the temperature switch. Afterward, we constructed a strain lacking the TCA cycle interventions of ita23 and replaced the native *icd* promoter by two versions of the temperature‐controlled Lambda promoter. We analyzed the behavior of the strains at 30 and 37°C and finally performed fed‐batch cultivations to study the effect of the resulting two‐stage process on the volumetric productivity compared to a single‐stage process. Indeed, compared to the one‐stage process, we significantly improved the peak productivity by 48% and the overall productivity by 22% after 120 hr and identified the glucose uptake rate in the production phase as major target for further productivity improvements.

## 2 METHODS

### 2.1 Strains and strain construction

The strains are listed in Table 1. Gene deletions or replacements were made by homologous recombination (Datsenko & Wanner, 2000) with pKD3 or pKD3_Ptemp as template, or by P1 transduction (Thomason, Costantino, & Court, 2007).

**Table 1:** Strains and plasmids

Strains or Plasmids	Description	Reference
Strains		
MG1655	Wild type	FRB426 (Blattner lab)
BW25141	*lacI^q^ rrnB_T14_ ΔlacZ_WJ16_ hsdR514 ΔaraBAD_AH33_ ΔrhaBAD_LD78_*	Datsenko and Wanner (2000)
BJH38	MG1655 *ΔaceA Δpta ΔpykF ΔpykA*	This study
BJH48	MG1655 *ΔPicd_untranslReg:: CAM_CI857_p*_R_ *_p*_L_ *_synthRBS*	This study
BJH58	MG1655 *ΔaceA Δpta ΔpykF ΔpykA ΔPicd_untranslReg::CAM_CI857_p*_R_ *_p*_L_ *synthRBS*	This study
ita23	MG1655 *ΔaceA ΔsucCD ΔpykA ΔpykF Δpta ΔPicd::cam_*P2 pCadCs	Harder et al. (2016)
ita32	MG1655 *ΔaceA Δpta ΔpykF ΔpykA* pCadCs	This study
ita35	MG1655 *ΔaceA Δpta ΔpykF ΔpykA ΔPicd::CAM_ CI857_p*_R_ *_p*_L_ pCadCs	This study
ita36A	MG1655 *ΔaceA Δpta ΔpykF ΔpykA ΔPicd::CAM_ CI857_p*_R_ pCadCs	This study
Plasmids		
pCadCs	BBa_J23100_*cadA_gltA_*BBa_0010, KanR, ColE1 origin	Harder et al. (2016)
pPL451	*CI857*, *p*_R_, *p*_L_	Love, Lilley, and Dixon (1996)
pKD46	*ParaB_gam_bet_exo*	Datsenko and Wanner (2000)
pKD3	*FRT_cam_FRT*	Datsenko and Wanner (2000)
pKD3_Ptemp	*FRT_cam_FRT_ CI857_P_R__P_L_*	This study
pKD3_P1	*FRT_cam_FRT_*BBa_J23117	This study

untranslReg: space between transcription and translation starting site; synthRBS: synthetic ribosome binding site (AGGAGGACAGCT); BBa_J23100, BBa_J23117: promoter from Anderson promoter collection (parts.igem.org); BBa_0010, terminator (parts.igem.org).

To construct the plasmid pKD3_Ptemp, the *CI857_p_R__p_L_‐*region from pPL451 was amplified using primers *PPL3_Ass* and *PPL4_Ass*, and the plasmid pKD3_P1 was amplified by primers *pKD3_AssPT_2_fw* (including a synthetic ribosome binding site) and *pKD3_Ass_PT_2_rv*. The PCR products were annealed by the Gibson assembly kit from *New England Biolabs* and transformed into BW25141. The plasmid pKD3_P1 was constructed as previously reported (Harder et al., 2016) using primers *Pkd3_P_rv* and *Pkd3_P1_fw*. Primer sequences for the deletion of the genes *aceA*, *pykA*, *pykF*, and *pta* are described in Harder et al. (2016) and additional primers are summarized in Table 2.

**Table 2:** Primers used in this study

Primer	Nucleotide sequence	Used for strain/plasmid
PPL3_Ass	AAGGAGGATATTCATATGGACCATGATTACGAATTGCC	pKD3_Ptemp
PPL4_Ass	GGAATTAAGCTGTCCTCCTGAGTTAACCTCCTTAGGATCC	pKD3_Ptemp
pKD3_AssPT_2_rv	TCCATATGAATATCCTCCTTAG	pKD3_Ptemp
pKD3_AssPT_2_fw	C**AGGAGGACAGCT**TAATTC	pKD3_Ptemp
Pkd3_P_rv	TACGGATCC**AGGAGGACAGCT**TAATTCCCATGTCAGCCGTTAAGTG	pKD3_P1
Pkd3_P1_fw	ACTGGATCCGCTAGCACAATCCCTAGGACTGAGCTAGCTGTCAATCCATATGAATATCCTCC	pKD3_P1
D_Picd_P2_fw	AAAGAAGTTTTTTGCATGGTATTTTCAGAGATTATGAATTGCCGCATTATGTGTAGGCTGGAGCTGCTTC	BJH48/BJH58/ita35/ita36
D_Picd_P2_rv	TGCAGGGTGATCTTCTTGCCTTGTGCCGGAACAACTACTTTACTTTCCATAGCTGTCCTCCTGGATCCGC	BJH48/BJH58
icd_nativeRBS_rv	TGCGATACGGATGCTTTAGAGCAATTTTTTGTTAATGATTTGTAATTGGCGGATCCCAATGCTTCGTTTC	ita35
icd_PR_nativeRBS	TGCGATACGGATGCTTTAGAGCAATTTTTTGTTAATGATTTGTAATTGGCTTAGCTGTCTTGGTTTGCCC	ita36
icd_fw_real	ACCCGAACACTGGCAAAGAGA	
icd_rv_real	TGCCAGGGCGTCAGAAATGTA	
infA_fw_real	CCATGTTCCGCGTAGAGTTAGA	
infA_rv_real	AGGTCGTACGGGGTCAGTTCAA	
recA_fw_real	CGCTTGGGGCAGGTGGTCT	
recA_rv_real	TGCAGCGTCAGCGTGGTTTT	
rpoD_fw_real	TCTGCGTATGCGTTTCGGTATC	
rpoD_rv_real	ACGGCTCGGGTGACGCAGTT	
ybhC_fw_real	GTCGCGGCGCAGTGGTGTT	
ybhC_rv_real	ACGGCTGTTTACGGCGAGGAA	

Sequence in bold: ribosome binding site. Underlined sequence: homologous to genome of *E. coli*.

### 2.2 Media and cultivation

For shake flask cultivations, if not stated otherwise, all strains were grown at appropriate temperature on solid or liquid LB_0_ medium (10 g/L tryptone, 5 g/L yeast extract, and 5 g/L NaCl). For strains BJH58, ita35 and ita36A additionally 2 g/L glucose and 2.5 mM CaCl_2_ were added. For the shake flask cultivations, a first preculture in LB_0_‐medium was conducted at 37°C, the second preculture in Minimal Medium (MM) (Causey, Zhou, Shanmugam, & Ingram, 2003) at the respective starting temperature (37, 30, or 28°C) of the main culture. All main cultures were performed with MM with 4 g/L glucose. Antibiotics were added at concentrations of 0.025 g/L for kanamycin, 0.1 g/L for ampicillin, and 0.025 g/L for chloramphenicol, respectively. The shake flask cultivations were performed at least in triplicates.

For the bioreactor cultivation strain ita36A grown on Tanaka agar plates with glucose as carbon source (TN plates; Tanaka, Lerner, & Lin, 1967) was directly inoculated into MM medium. For the one‐stage process the cells were grown for 8 hr at 37°C and then shifted to 28°C for 16 hr. The preculture for the two‐stage process was incubated for 24 hr at 37°C. The MM medium was slightly modified for the bioreactor cultivation by increasing the MgSO_4 _· 7H_2_O concentration to 0.5 g/L, glucose to 30 g/L, and by addition of Antifoam 204 (Sigma‐Aldrich, Darmstadt, Germany) to a final concentration of 0.005 %. The pH was maintained at 6.9 by addition of 2 M NaOH (one‐stage: 0–49 hr; two‐stage: 0–24 hr) or 5 M NaOH. Medium for the manual feed pulses contained 300 g/L glucose, 52.5 g/L (NH_4_)_2_SO_4_, and 0.005% antifoam 204 as well as 0.025 g/L kanamycin. Fed‐batch cultivations were carried out in 1 L Multifors‐bioreactors (Infors) with a starting volume of 0.4 L and an agitation of 550 rpm which was increased during the cultivation to maintain dissolved oxygen concentration at levels above 30%. The temperature was set to 28°C. To reach a high cell density, the ammonium concentration was increased by a manual feed of 2 g (NH4)_2_SO_4_ (one stage: 47 hr, two‐stage: 10 hr).

### 2.3 Analytics, enzyme assays, calculation of rates

Extracellular metabolites were quantified by HPLC (Harder et al., 2016) or by using enzyme kits from Megazyme. Enzyme activities and yields were determined as previously reported (Harder et al., 2016). Gene expression was measured in two independent biological samples of BJH58 and three samples of MG1655, ita35, and ita36A at the respective temperatures. Two mRNA aliquots were transcribed to cDNA, pooled, and then measured three times by qRT‐PCR. The procedure of RNA isolation, reverse transcription and qRT‐PCR is described in detail in Steinsiek, Stagge, and Bettenbrock (2014). The sequences of the primers for *icd* and for the reference genes *infA*, *rpoD*, *recA*, and *ybhC* are given in Table 2.

Metabolic flux balance analysis was performed with the Matlab Toolbox *CellNetAnalyzer* (Klamt, Saez‐Rodriguez, & Gilles, 2007) using our metabolic model for itaconic acid production (Harder et al., 2016).

## 3 RESULTS AND DISCUSSION

### 3.1 Control of growth rate by dynamic regulation of isocitrate dehydrogenase

**Figure 2:**
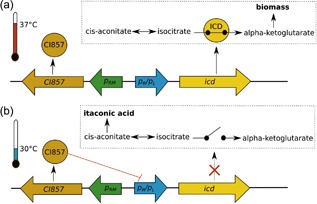
Dynamic control of *icd* expression. (a) The repressor *CI857* is inactive at 37°C allowing expression of *icd* for an active TCA cycle and biomass formation. (b) At 30°C, the repressor becomes active, binds to the promoters, (*p_R_*/*p_L_*) and, therefore, blocks transcription of *icd* and thus the TCA cycle redirecting flux to itaconic acid

We have shown in our previous study that the inhibition of isocitrate dehydrogenase (ICD), in combination with several other knockouts in the central metabolism (strain ita23 in Figure 1), is a key target to reach a high itaconic acid yield with *E. coli* (Harder et al., 2016). Due to high product formation, ita23 grew with a low growth rate and, therefore, the productivity of the fed‐batch process was limited by the low biomass concentration. To increase the growth rate and to allow biomass formation without glutamate supplementation we decided to decouple biomass and product synthesis by dynamically regulating the *icd* expression. Control of genes expressed by the bacteriophage λ promoters (*p*_R_, *p*_L_) using the temperature‐sensitive repressor *CI857* has been widely used for dynamically activating the production of recombinant proteins in *E. coli* simply by increasing the temperature (Valdez‐Cruz, Caspeta, Pérez, Ramírez, & Trujillo‐Roldán, 2010). We considered this system attractive for our desired regulation of the ICD, although in the reverse direction: in our case ICD is needed to be active during cell growth (favored by an inactive *CI857* at 37°C) and inactive in the second phase of itaconic acid production (favored by an active *CI857* at 30°C). The logic of our intended dynamic switch is shown in Figure 2. To first demonstrate that we can control cell growth by dynamic regulation of ICD by this temperature‐dependent switch we proceeded as Zhou, Deng, Cui, Liu, and Zhou (2015) and replaced the promoter including the untranslated region upstream of the *icd* gene by the Lambda promoters *p*_R_ and *p*_L_ and a synthetic ribosome binding site for translation of *icd*. The repressor *CI857*, controlling the expression from *p*_R_ and *p*_L_, is expressed from the promoter for repressor maintenance (*p*_RM_). *CI857* autoregulates its expression by activating the expression from *p*_RM_ when bound to operator region 2 (*O*_R_2) of *p*_R_ and, at high concentrations, represses the expression when bound to *O*_R_3 (Meyer, Maurer, & Ptashne, 1980). The derived strain BJH48 showed the expected behavior, with a high growth rate (0.43 hr^−1^ ± 0.04) at 37°C and a sharply decreased growth rate (0.05 hr^−1^ ± 0.00) at 30°C. BJH48 showed nearly no ICD activity at 30°C (0.008 U/mg Protein) compared to the wild type activity of 1 U/mg Protein. This demonstrates that the Lambda phage promoters can be used to dynamically knock‐down the *icd* gene.

### 3.2 Dynamic regulation of itaconic acid production

To dynamically control itaconic acid production, we first constructed a strain ita32 with all knockouts of ita23 except for *icd* and *sucCD* (Figure 1). This strain (ita32) showed the wild type (MG1655) growth behavior at 30°C with increased glutamate production (Table 3). This is most likely a consequence of the imbalanced flux through the TCA cycle due to the NADH insensitivity of the citrate synthase (CS) of *Corynebacterium glutamicum* which is co‐expressed with the cis‐aconitate decarboxylase (CAD) via the pCadCS plasmid in ita32 (Table 1). At 37°C, ita32 still grew with a high growth rate of 0.52 hr^−1^ and, therefore, presents a suitable target strain for dynamic process regulation. The excreted glutamate could be reused in the production phase. Without the pCadCS plasmid, the strain (BJH38) was nearly identical to the wild type except for a reduced production of acetate.

**Table 3:** Shake flask cultivations at high (37°C) and low (28°C/30°C) temperatures

					Yield (mol/mol [glucose])			
Strain	Temperature/preculture condition	*μ* [hr^−1^]	*r*_glucose _ [mmol/gDW/hr]	*Y*_biomass/glucose _ [g/mmol]	Itaconate	Glutamate	Acetate	Pyruvate
MG1655	30°C Pc^L,MM^	0.36 ± 0.01	4.36 ± 0.27	0.082 ± 0.003	0	0.00 ± 0.00	0.34 ± 0.00	0 ± 0
	37°C Pc^L,MM^	0.71 ± 0.01	7.62 ± 0.08	0.095 ± 0.004	0	0.01 ± 0.02	0.34 ± 0.04	n.m.
BJH38	37°C Pc^L,MM^	0.69 ± 0.01	7.64 ± 0.71	0.093 ± 0.006	0	0.02 ± 0.01	0.06 ± 0.01	n.m.
ita32	30°C Pc^L,MM^	0.34 ± 0.03	4.74 ± 0.38	0.074 ± 0.010	0.01 ± 0.01	0.17 ± 0.06	0.06 ± 0.01	0 ± 0
	37°C Pc^L,MM^	0.52 ± 0.05	8.53 ± 0.43	0.062 ± 0.01	0.00 ± 0.00	0.30 ± 0.01	0.01 ± 0.01	0 ± 0
BJH58	37°C Pc^L,MM^	0.14 ± 0.02	n.m.	n.m.	n.m.	n.m.	n.m.	n.m.
ita35	30°C Pc^L,MM^	0.11 ± 0.01	3.26 ± 0.80	0.034 ± 0.001	0.66 ± 0.07	0.00 ± 0.00	0.08 ± 0.01	0.17 ± 0.07
	37°C Pc^L,MM^	0.50 ± 0.03	8.98 ± 0.62	0.060 ± 0.005	0	0.37 ± 0.13	0.01 ± 0.01	0
ita36A	30°C Pc^L,MM^	0.12 ± 0.02	3.25 ± 0.70	0.034 ± 0.000	0.66 ± 0.05	0.00 ± 0.00	0.06 ± 0.02	0.17 ± 0.04
	37°C Pc^L,MM^	0.46 ± 0.12	7.05 ± 0.31	0.067 ± 0.031	0 ± 0	0.21 ± 0.16	0.01 ± 0.01	0 ± 0
	37°C Pc^MM^	0.53 ± 0.07	6.86 ± 0.06	0.078 ± 0.011	n.m.	0.11 ± 0.12	0.08 ± 0.01	n.m.
	30°C Pc^MM^	0.14 ± 0.01	3.82 ± 0.12	0.034 ± 0.00	0.62 ± 0.03	0 ± 0	0.10 ± 0.03	0.2 ± 0.03
	28°C Pc^MM^	0.12 ± 0.00	3.73 ± 0.03	0.031 ± 0.00	0.69 ± 0.01	n.m.	0.10 ± 0.01	0.17 ± 0.01

Pc^L,MM^: two precultures were used, one in LB_0_, followed by one in MM; Pc^MM^: only one preculture in MM was used; n.m., not measured.

**Figure 3:**
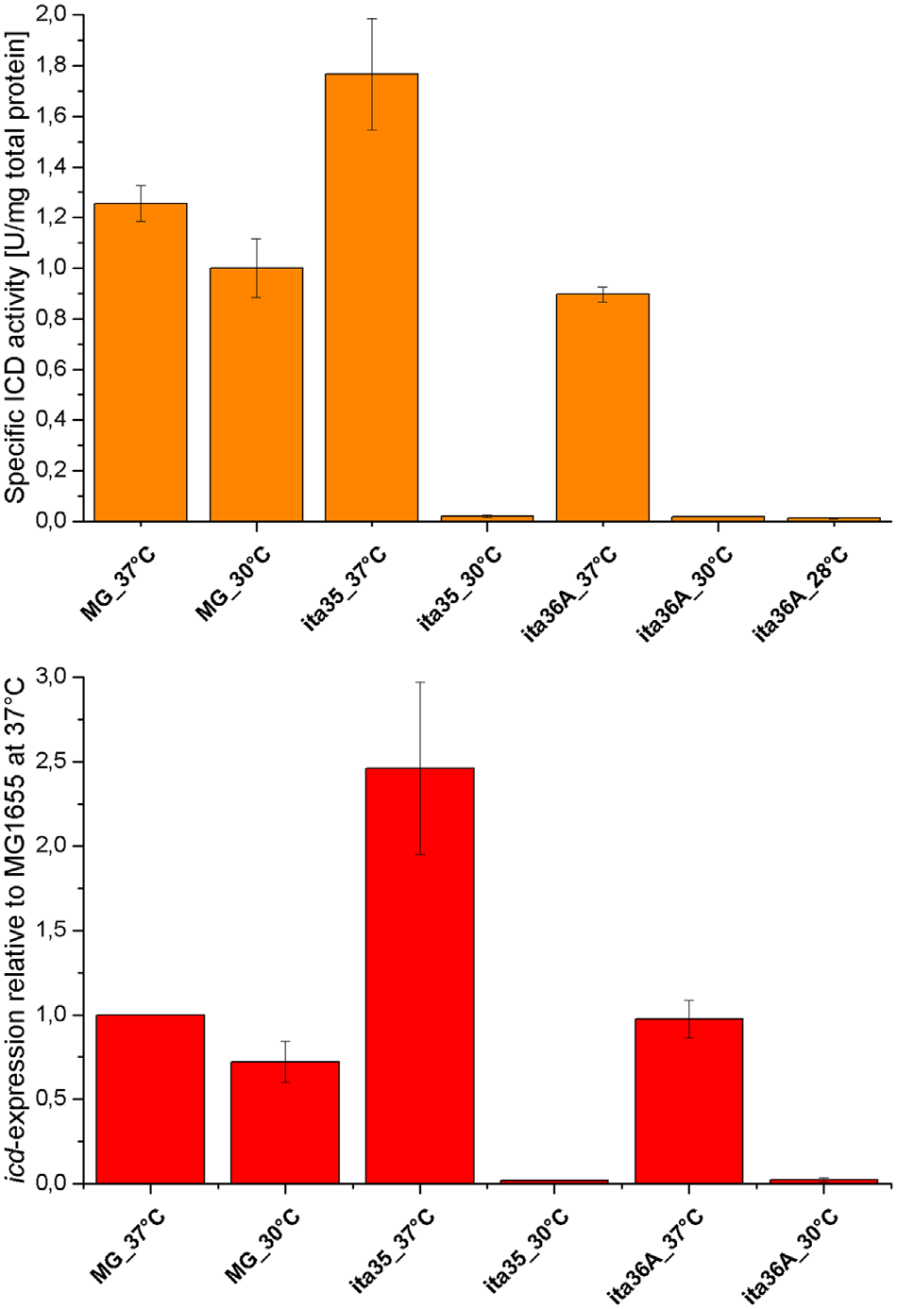
Top: Specific activities of the isocitrate dehydrogenase (ICD). The activity was normalized to the total protein content in the sample. Bottom: Relative expression of *icd* normalized to the wild type at 37°C. Error bars represents the standard deviation of two independent biological samples (for the activity) and three independent biological samples (for the expression level). The expression level of ita36A at 28°C was not determined

Analogous to BJH48, we replaced in BJH38 186 bp directly upstream of the *icd* gene, including the natural promoter and ribosome binding site (RBS), by the Lambda promoters (*p*_R_ and *p*_L_) and a synthetic RBS. The derived strain BJH58 grew with a low growth rate of 0.14 hr^−1^ at 37°C resulting from low expression of *icd* (16 times lower than in the wild type) and, therefore, low ICD activity (0.02 ± 0.01 U/mg total protein). The expression of genes is often not only affected by the promoters alone but also by translation efficiency and mRNA stability. It has been shown that the sequence between transcription and translation start site has a significant impact on the expression level, because it can include regulatory elements or affect mRNA stability (De Mey et al., 2010). We hypothesized that the untranslated region of the *icd* gene is important for mRNA stability and has to be conserved to provide a sufficient ICD flux for fast growth of our target strain BJH38. To test our hypothesis, we placed the Lambda promoters upstream of the transcriptional initiation site, replacing the natural promoter but conserving the 115 bp region upstream of the *icd* gene including its natural RBS. Indeed, the high growth rate of the wild type was maintained in the respective strain (data not shown). With pCadCs, this strain (ita35) had a similar growth rate as ita32 at 37°C with slightly increased glutamate production (Table 3). At 30°C ita35 had the desired phenotype of growth‐coupled itaconic acid synthesis with a yield of 0.66 mol/mol and a growth rate of 0.11 hr^−1^. The expression of *icd* at 30°C was highly downregulated, 57 times lower than in the wild type at 37°C. We found that the expression and activity of the ICD in ita35 at 37°C were significantly increased compared to the wild type (Figure 3) and, therefore, tested another strain (ita36A) with *icd* expression from only one Lambda promoter (*p_R_*). This strain exhibited reduced ICD activity (Figure 3) and a slightly reduced growth rate at 37°C, but had an identical phenotype at 30°C compared to ita35. In some cases, when cells reached a low cell density in the second preculture in MM after overnight incubation, the growth of ita36A at 37°C was delayed by a prolonged lag‐phase or even slow growth (*μ* < 0.2 hr^−1^) in the main culture. In the LB_o_ preculture, we observed exceptional long cells, indicating cell division defects. Therefore, we directly inoculated a MM pre‐culture (Pc^MM^) from TN agar plates (Table 3). With Pc^MM^ the glutamate production at 37°C was further decreased while the growth rate was increased to 0.53 hr^−1^. The production at 30°C was similar and was further increased to 0.69 mol/mol by lowering the temperature to 28°C. Surprisingly, the strain still grew at 30°C with 0.14 hr^−1^ and at 28°C with 0.12 hr^−1^, although nearly no ICD activity was measured in extracts (0.02 and 0.01 U/mg total protein, Figure 3). It has to be mentioned that this activity is comparable to strain ita23 (Harder et al., 2016) which was auxotrophic for glutamate. So the main difference between the strains at 30°C is the *sucCD* knockout in ita23. In ita36A, succinyl‐CoA can be regenerated from succinate and, therefore, a lower flux through the ICD seems to be sufficient to provide precursors for growth.

### 3.3 Two‐stage bioreactor cultivation increases titer and volumetric productivity

In the last step, we aimed to utilize our genetic switch of the ICD in strain ita36A to establish a two‐stage process for itaconic acid production. The ultimate goal was to increase our previously reported overall volumetric productivity of 0.27 g/L/hr and peak productivity of 0.45 g/L/hr (strain ita23) after 120 hr (Harder et al., 2016).

With protein half‐lives in the order of hours (Venayak et al., 2015), a changed expression level is not immediately represented by the phenotype. When we cultivated ita36A in shake flasks at 28°C, inoculated from a 37°C preculture, it took around 12 hr to switch to itaconic acid production. Hence, we decided to use this time delay as growth phase in our two‐stage bioreactor process. To compare the different process setups, we incubated the preculture for the one‐stage process (growth‐coupled itaconic acid synthesis) for 8 hr at 37°C followed by 16 hr incubation at 28°C, whereas the preculture for the two‐stage process was incubated at 37°C for 24 hr. The temperature in the bioreactor was set to 28°C for both cases and the pH was maintained at 6.9 by addition of NaOH. The here used approach is thus not a classical two‐stage process where an external or internal signal during the cultivation leads to the shift to the production phase. However, the two‐stage character was nevertheless achieved by preparing the preculture for a high growth rate at the initial stage (0–12 hr) of the cultivation while the low temperature over the whole runtime of the process guarantees a (delayed) switch to the production stage.

**Figure 4:**
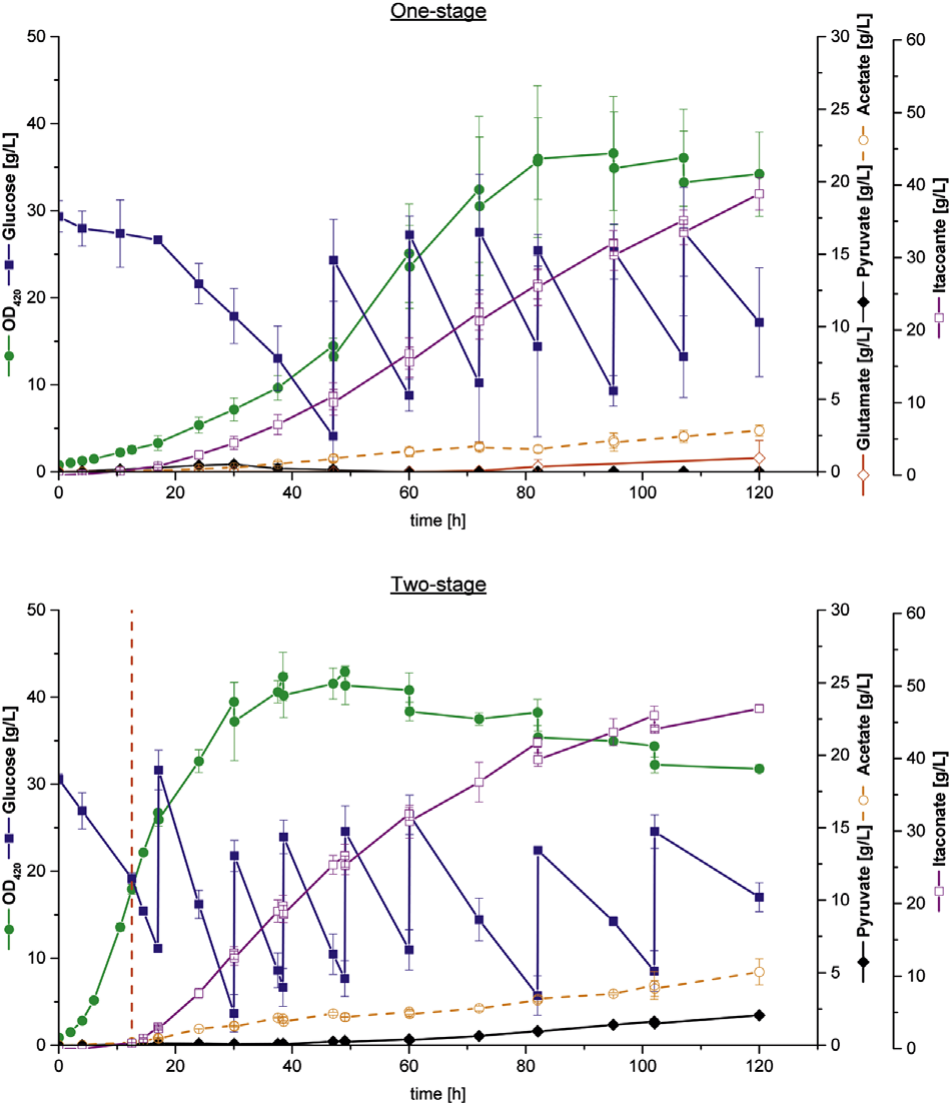
One‐stage and two‐stage bioreactor cultivations of ita36A at 28°C. Both processes differed with respect to their preculture conditions: the one‐stage preculture was conducted at 28°C (optimal for production), the two‐stage preculture at 37°C (optimal for growth). This enabled fast growth of ita36A at the initial stage of the two‐stage process (0–12 hr). The point where the transition from growth to production phase occurred is marked by the red dashed line. The addition of feed solution is marked by a sharp increase in glucose concentrations. The one‐stage process was conducted as triplicate, the two‐stage process as duplicate. Error bars represents the standard deviation of the independent biological samples. Glutamate was not produced in the two‐stage cultivation

**Figure 5:**
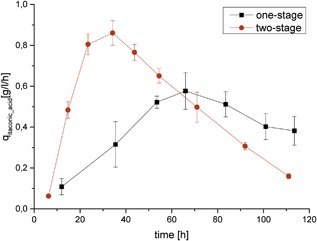
Dependence of the volumetric productivity of Ita36A on the cultivation time and process design. Error bars represent the standard deviation of the independent biological samples

In the two‐stage process, ita36A grew in the first 12 hr with a maximal growth rate of 0.25 hr^−1^ with nearly no itaconic acid production and then switched to a growth rate of 0.1 hr^−1^ which is similar to the maximal growth rate of the one‐stage process (Table 4) and started to produce itaconic acid (red dashed line Figure 4). After 30 hr, 8 g/L biomass was produced whereas the biomass concentration of 1.5 g/L in the one‐stage process was significantly lower (Figure 4). The high biomass concentration in the two‐stage process enabled fast production of 32 g/L itaconic acid within 60 hr with a peak productivity of 0.86 g/L/hr. Then growth stopped and the productivity decreased because of reduced glucose uptake rates (Figure 5). Apparently, when cells stopped growing, they only took up glucose to provide ATP for maintenance processes. Indeed, with the measured glucose uptake rates and product formation rates of the last feed we estimated with a metabolic model maximal ATP production rates for the stationary phase of 3.95 mmol/gDW/hr in the one‐stage process and 2.71 mmol/gDW/hr in the two‐stage process. These rates are close to the maintenance value of 3.15 mmol/gDW/hr used in the latest genome‐scale metabolic model of *E. coli* (Orth et al., 2011), supporting the hypothesis that the ATP demand is the main driving force for itaconic acid production in the stationary phase.

**Table 4:** Comparison of bioreactor cultivations of ita36A at 28°C

	Batch			Feed			Overall (0–120 hr)		
	ita23 (one‐stage)	ita36A (one‐stage)	ita36A (two‐stage)	ita23 (one‐stage)	ita36A (one‐stage)	ita36A (two‐stage)	ita23 (one‐stage)	ita36A (one‐stage)	ita36A (two‐stage)
*μ*_max_ [hr^−1^]	0.07 ± 0.00*	0.08 ± 0.00	0.25 ± 0.01	0.02 ± 0.00*	0.05 ± 0.02	0.03 ± 0.00			
*Y*_itaconic_acid_ [mol/mol]	0.54 ± 0.00*	0.58 ± 0.06	0.19 ± 0.01	0.71 ± 0.07*	0.60 ± 0.12	0.7 ± .02	0.68 ± 0.04*	0.62 ± 0.11	0.62 ± 0.00
*Y*_acetate_ [mol/mol]	0.02 ± 0.01*	0.10 ± 0.01	0.06 ± 0.01	0.03 ± 0.00*	0.09 ± 0.02	0.15 ± 0.03	0.04 ± 0.00*	0.10 ± 0.02	0.14 ± 0.03
*Y*_glutamate_ [mol/mol]	−0.10 ± 0.01*	0.00 ± 0.00	0.00 ± 0.00	−0.08 ± 0.01*	0.03 ± 0.04	0.00 ± 0.00	−0.08 ± 0.01*	0.01 ± 0.01	0.00 ± 0.00
*r*_glucose_ [mmol/gCDW/hr]	3.13 ± 0.42*	2.94 ± 1.10	3.48 ± 1.42	1.39 ± 0.34*	0.99 ± 0.45	0.85 ± 0.39			
Itaconic acid titer [g/L]	9.2 ± 1.0*	10.9 ± 1.9	2.96 ± 0.20				32 ± 1*	38.8 ± 2.20	46.9 ± 0.5
*q*_itaconic_acid_ [g/L/hr]	0.22 ± 0.02*	0.24 ± 0.03	0.17 ± 0.01	0.38 ± 0.08*	0.38 ± 0.03	0.43 ± 0.00	0.27 ± 0.01*	0.32 ± 0.02	0.39 ± 0.00
Biomass [g/L]	2.0 ± 0.2*	3.0 ± 0.2	5.6 ± 0.1				3.4 ± 0.0*	7.2 ± 1.0	6.7 ± 0.1

One‐stage: preculture 28°C; two‐stage: preculture 37°C.

* For ita23, we used the bioreactor cultivation data from our previous study (Harder et al., 2016).

If we now compare the two‐stage process with the one‐stage process after 120 hr, we can see a significant increase in the overall productivity by 22% and a 48% higher maximal productivity (Figure 5) with the decoupled itaconic acid production process. Compared to strain ita23 (Harder et al., 2016) titer and peak productivity were even increased by 46% and 91%, respectively. Clearly, the comparison of the overall productivity depends on the chosen reference time point. At earlier time points, the relative performance gain is even higher (e.g., 62% [instead of 22%] productivity improvement after 82 hr). Later in the process, the advantage of the two‐stage process gets reduced as specific glucose uptake rates and itaconic acid production rates decrease in the non‐growth phase.

## 4 CONCLUSION

Expression of genes by the Lambda promoters (*p*_R_ and *p*_L_) can be controlled by the temperature dependent repressor *CI857* which is inactive at high temperatures (Lieb, 1966). The advantage of this control system is that it can be regulated simply by changing the temperature and does not require an expensive inducer, a pre‐condition for industrial applications. The dynamic activation of the lactate production pathway in an oxygen‐limited phase at 42°C with this genetic switch successfully increased d‐lactate production by *E. coli* (Zhou et al., 2012). Although it has been shown that the Lambda promoters can be used in the opposite direction to switch genes off (Cho et al., 2012), they have not been used so far in this way to improve cell factory performance. Here we demonstrated that we can control the TCA cycle and the growth rate by temperature‐dependent expression of the isocitrate dehydrogenase. The ICD flux needed for fast growth depends on the strain background and can be optimized by improving the promoter or the ribosome binding site. The next step was to develop a two‐stage process for itaconic acid production. Strains for two‐stage processes should fulfill three criteria in the first (growth) phase: (1) fast growth; (2) high biomass yield; and (3) low amounts of by‐products. Indeed, the selected starting strain for the dynamic regulation of itaconic acid production ita32 (MG1655 Δ*aceA* Δ*pta* Δ*pyA* Δ*pykF* pCadCs) grew with a fast rate of 0.52 hr^−1^, synthesized biomass with a yield of 0.062 mmol/gDW/hr and produced some glutamate as by‐product. Based on ita32, we constructed a dynamic itaconic acid production strain ita36A with temperature dependent expression of the isocitrate dehydrogenase from *p*_R_. Under optimized conditions this strain grew comparable to ita32 at 37°C with reduced glutamate production and switched, as desired, to high yield itaconic acid production at 28°C with a reduced growth rate of 0.12 hr^−1^. The switching time of around 12 hr, resulting from slow degradation of the proteins, might be further decreased by adding degradation tags to the C‐terminus of the isocitrate dehydrogenase or by engineering the ICD for reduced activity at low temperature as described by (Cho et al., 2012) for the glyceraldehyde‐3‐phosphate dehydrogenase. We considered the time delay of the switch for the two‐stage bioreactor cultivation, inoculated with a preculture grown at 37°C, by directly starting with the temperature of the production phase (28°C). Compared to the one‐stage process (preculture at 28°C) the overall volumetric productivity after 120 hr was improved by 22% to 0.39 g/L/hr and an even better relative performance (62%) can be seen for earlier time points. Compared to our production strain ita23 the titer was improved by 46% and the peak volumetric productivity by 91%. In addition we could resolve the glutamate auxotrophy and the reached titer of 46.9 g/L is close to the recommended value of 50 g/L for commercialization of bioprocesses (Van Dien, 2013). This shows the further advantage of two‐stage processes: growth‐essential genes can be knocked‐down in the production phase without further medium supplementation. Generally, when designing two‐stage processes, one has to decide about the desired duration of the process, the target biomass and product yield, the target titer, and the target productivity (clearly, some of these parameters are not independent). Once these parameters have been fixed, it will be straightforward to calculate the optimal switching time point. For example, if desired, the growth phase of our two‐stage production process could be further extended by starting with a temperature of 37°C and then switching to 28°C at a later time point.

The observed reduced glucose uptake rate in the stationary (production) phase reduced the overall productivity of the long‐term cultivation process. To further increase the overall productivity it is thus necessary to maintain the high peak productivity during the second stage. In particular, despite the fact that lower temperatures reduce substrate uptake (even in the wild type; see Table 3), there is still a large potential for much higher productivities because the glucose uptake rate in the non‐growth phase did not exceed 1 mmol/gDW/hr. Consequently, cells have to be forced to high glucose uptake rates even when they are not growing. As the ATP maintenance demand seems to be the main driving force for itaconic acid production in this phase, ATP‐wasting strategies (Hädicke, Bettenbrock, & Klamt, 2015; Hädicke and Klamt, 2015; Liu, Kandasamy, Wurtz, Jensen, & Solem, 2016) could be used to increase substrate turnover. Besides, manipulation of regulatory networks (Michalowski, Siemann‐Herzberg, & Takors, 2017) and certain nutrient limitations (Chubukov & Sauer, 2014) might further increase uptake rates.

In contrast to naturally decoupled production processes mainly induced by nutrient limitations, our dynamic approach based on a temperature switch can generally be used to selectively knock‐down genes in *E. coli* in a second production phase, independent from the production pathway. We think that the identification and dynamic control of a metabolic node distinguishing between growth and production could become a general starting step in the rational design of efficient cell factories.
